# Two decades of astrocytes in neurovascular coupling

**DOI:** 10.3389/fnetp.2023.1162757

**Published:** 2023-04-03

**Authors:** Annamaria Lia, Alessandro Di Spiezio, Michele Speggiorin, Micaela Zonta

**Affiliations:** ^1^ Neuroscience Institute, National Research Council (CNR), Padua, Italy; ^2^ Department of Biomedical Sciences, University of Padua, Padua, Italy

**Keywords:** astrocytes, cerebral blood flow (CBF), calcium signal, neurovascular coupling (NVC), brain pathologies

## Abstract

The brain is a highly energy demanding organ, which accounts in humans for the 20% of total energy consumption at resting state although comprising only 2% of the body mass. The necessary delivery of nutrients to brain parenchyma is ensured by the cerebral circulatory system, through the exchange of glucose and oxygen (O_2_) at the capillary level. Notably, a tight spatial and temporal correlation exists between local increases in neuronal activity and the subsequent changes in regional cerebral blood flow. The recognized concept of neurovascular coupling (NVC), also named functional hyperemia, expresses this close relationship and stands at the basis of the modern functional brain imaging techniques. Different cellular and molecular mechanisms have been proposed to mediate this tight coupling. In this context, astrocytes are ideally positioned to act as relay elements that sense neuronal activity through their perisynaptic processes and release vasodilator agents at their endfeet in contact with brain parenchymal vessels. Two decades after the astrocyte involvement in neurovascular coupling has been proposed, we here review the experimental evidence that contributed to unraveling the molecular and cellular mechanisms underlying cerebral blood flow regulation. While traveling through the different controversies that moved the research in this field, we keep a peculiar focus on those exploring the role of astrocytes in neurovascular coupling and conclude with two sections related to methodological aspects in neurovascular research and to some pathological conditions resulting in altered neurovascular coupling.

## Introduction

The brain is populated by several cells, integrated in extracellular space and matrix to compose brain active milieu (Semyanov and Verkhratsky, 2021). All these components work in concert to ensure a proper cerebral function, contributing to processes such as development and maintenance of neuronal connectivity, information transfer and processing, metabolic supply, inflammatory responses and clearance of catabolites. A peculiar, dynamic unit operating in the brain milieu is the neurovascular unit (NVU), which comprises neurons, perivascular glia, mural cells, endothelial cells and extracellular matrix proteins interacting to guarantee blood brain barrier function and modulate cerebral blood flow (CBF).

Notably, the lack of energy storing in the cerebral tissue and the high level of energy consumption underlie the necessity of a continuous and controlled blood flow supply to the brain, in order to avoid permanent brain damage upon failure in the delivery of nutrients and oxygen. CBF regulation explicates at different levels. First, the flow needs to remain stable in spite of changes in the systemic blood pressure, a process known as cerebral autoregulation and mainly achieved through the myogenic reflex, in which smooth muscle cells modify cerebrovascular resistance to counterbalance pressure variations. This intrinsic regulation has been shown to be more efficient to compensate transient hypertension than hypotension (Brassard et al., 2017). An additional regulatory mechanism intervenes to adjust systemic arterial blood pressure and heart rate in case of a decrease of cerebral perfusion, as highlighted by recent studies supporting a role of astrocytes as intracranial baroreceptors, which sense hypoperfusion and promote the homeostatic control of brain blood flow through activation of the sympathetic system (Marina et al., 2020). Besides these mechanisms related to changes in blood flow pressure, a more local and tight bond links regional neural activity to CBF, in order to match, both temporally and spatially, the energy supply to metabolic demands. This fundamental relationship is named neurovascular coupling (NVC).

## Historical perspective: From 1880 to the 2000s

The initial evidence on NVC dates back to the end of 19th century, when the physiologist Angelo Mosso observed heartbeat pulsations in the exposed brain of two adults presenting head injuries. Thanks to his ingenious technical devices, he revealed a correlation between the magnitude of these pulsations and the grade of mental activities, concluding “We must suppose a very delicate adjustment whereby the circulation follows the needs of the cerebral activity. Blood very likely may rush to each region of the cortex according as it is most active” (Mosso, 1880). 10 years later, the pathologists Roy and Sherrington studied the regulation of CBF in animals and postulated the metabolic hypothesis, which links vascular supply to functional activity through a feedback mechanism initiated by chemical products of cerebral metabolism (Roy and Sherrington, 1890). Curiously, it was in the same years that Santiago Ramón y Cajal, looking at the morphological features of astrocytes in the architecture of brain milieu, imagined a role for “perivascular neuroglial cells” in the local dilatation of vessels, as a result of a putative mechanical movement of astrocyte endfeet (Cajal, 1895). The absence of adequate technical tools long represented the limiting step for an accurate study of regional blood flow. Convincing experimental advancements were indeed made through the years together with the development of new technological approaches, such as autoradiography combined to diffusible radioactive tracers in animals and humans (Freygang and Sokoloff, 1959; Lassen et al., 1978). After more than one century from the observations of Mosso, the development of functional magnetic resonance imaging (fMRI) in humans ultimately confirmed with high temporal and spatial resolution the main concept of functional hyperemia, linking local CBF to neuronal function (Raichle and Mintun, 2006).

In parallel, the research on the cellular and molecular mechanisms involved in this coupling was taking its early steps. The first study on the role of astrocytes in NVC (Zonta et al., 2003a) investigated the process of vasodilatation evoked by electrical neuronal stimulation *ex vivo* and by sensory stimulation *in vivo,* demonstrating a central role for neurotransmitter-dependent activation in astrocytes of metabotropic glutamate receptors (mGluRs). These G-protein coupled receptors (GPCRs) are linked to the intracellular pathway of Ca^2+^ release from endoplasmic reticulum. Consistently, perfusion with mGluR agonist or direct activation of Ca^2+^ increases in patched astrocytes was sufficient to trigger arteriole dilatation in cortical slices. All protocols used to induce vasodilatation *ex vivo* were significantly dependent on the release of cyclooxygenase (COX) products, most likely prostaglandin E2, which is released by astrocytes in a Ca^2+^-dependent process (Zonta et al., 2003b). The involvement of astrocytes in NVC was supported by another study performed in the somatosensory cortex (SSCx) of anesthetized adult mice, where Ca^2+^ uncaging in astrocyte elicited a local hyperemic response that was sensitive to specific COX-1 inhibitors. Similarly, triggering neuronal activity *via* extracellular electrical stimulation induced Ca^2+^ responses in astrocytes and resulted in arteriole vasodilatation, significantly reduced by mGluR antagonists and COX-1 inhibitors (Takano et al., 2006).

**FIGURE 1:**
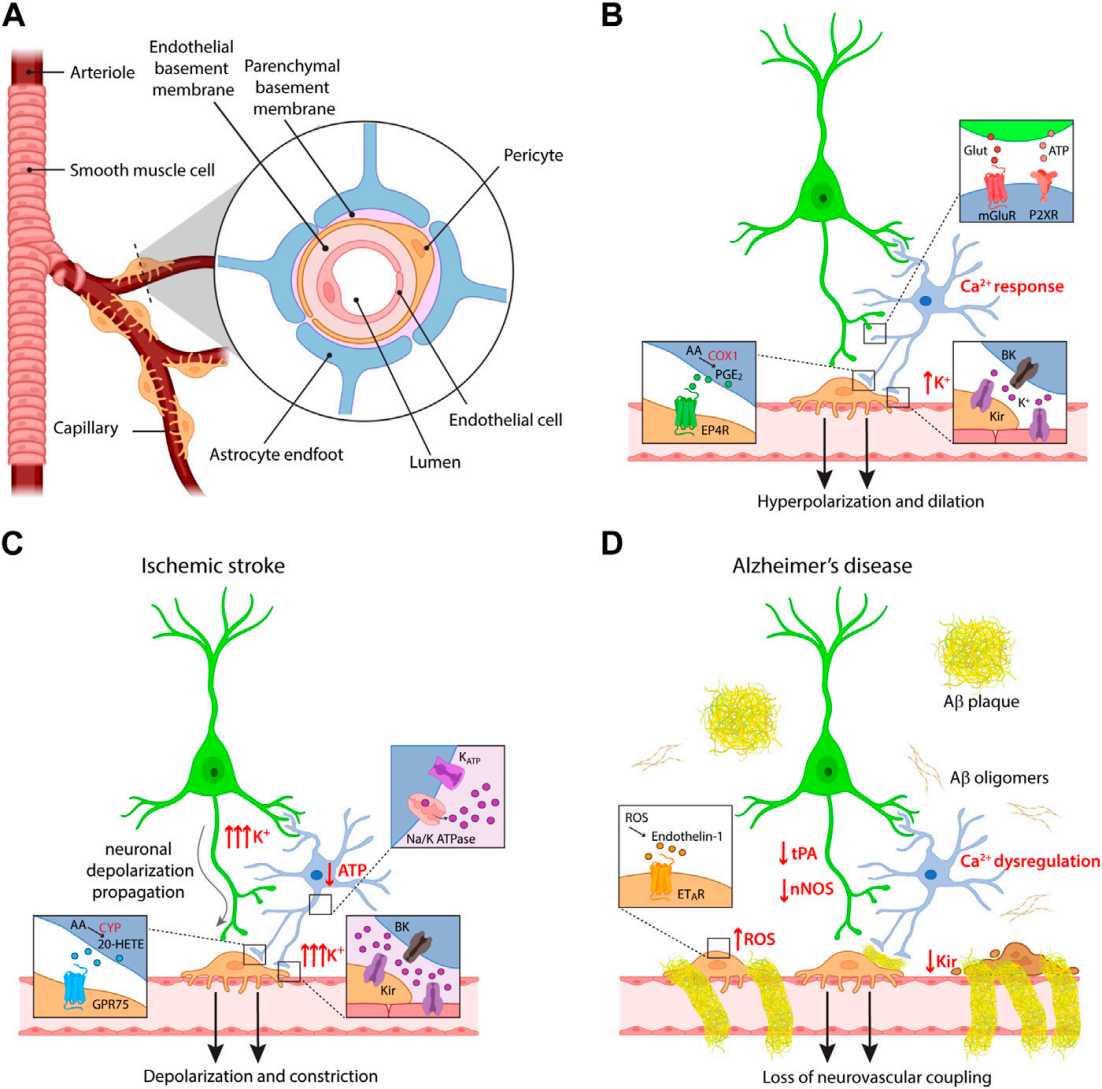
Cellular and molecular mechanisms of neurovascular coupling. **(A)** Schematic of the components of the neurovascular unit at the capillary level. **(B)** Major pathways of astrocyte contribution in NVC upon physiological conditions. **(C,D)** Dysregulation of neurovascular coupling upon different pathological conditions, i.e., ischemic stroke and Alzheimer’s disease. Some of the insets provided in panel **(B)** are omitted for clarity in these panels. For the same reason, the reduction in ATP production and the effects on K^+^ fluxes are indicated only in the astrocyte, but involve also the other cells.

In both works, a residual vasodilatation persisted after inhibition of COX pathway. The nature of the pathway mediating this residual component was disclosed by a concomitant study in cortical slices, which demonstrated that neuronal stimulation induces in astrocyte endfeet the opening of big potassium (BK) channels, large conductance calcium-activated K^+^ channels that mediate K^+^ efflux. The consequent increase in extracellular K^+^ concentration ([K^+^]_o_) activates inward rectifier potassium (Kir) channels in smooth muscle cells (SMCs), resulting in cell hyperpolarization and vasodilatation. Importantly, the concurrent inhibition of BK channels and of COX activity resulted in a complete blockade of neuronal activity-dependent vasodilatation (Filosa et al., 2006). Figures 1A, B summarize the molecular pathways described by these studies for astrocyte contribution to NVC.

## Dilatation vs constriction

From these seminal works onwards, different lines of controversy raised to dispute the involvement of astrocytes in NVC. The first argument concerned the polarity of the vascular response, since astrocyte activation in brain slices was found to evoke also vasoconstriction (Mulligan and MacVicar, 2004; Metea and Newman, 2006). In depth investigation of the factors affecting dilatation/constriction balance revealed that the polarity of vascular response to astrocyte stimulation depends on the resting arteriolar tone (Blanco et al., 2008) and on the metabolic state of the cerebral tissue (Gordon et al., 2008). Indeed, a physiological range of 30%–40% myogenic tone favors vasodilatation, while a lower tone, such as in brain slices lacking blood circulation, promotes constriction upon the same vasoactive stimuli, e.g., mGluR activation or high [K^+^]_o_. On the other hand, the enzymatic reactions controlling the synthesis of vasoactive metabolites from arachidonic acid (AA) are sensitive to oxygen concentration, resulting in a shift of vascular response towards dilatation at physiological O_2_ levels and towards constriction at higher levels. It is noteworthy that standard procedures of O_2_ equilibration in slice experiments result in supra-physiological oxygenation, thus making dilatation more difficult to observe. Beyond affecting AA metabolism, oxygenation levels influence NVC also by regulating glycolysis, in that O_2_ consumption accompanying neuronal activity boosts anaerobic glycolysis. The consequent increase in the production and release of lactate leads to a reduction of prostaglandin clearance *via* the prostaglandin/lactate transporter, enhancing vasodilatation, while the lower production of ATP results in an increased availability of the vasodilator adenosine (Attwell et al., 2010).

## A role for astrocyte Ca^2+^

Along the wave of a larger criticism on the physiological role of gliotransmission, different publications questioned the dependency of vasodilatation on astrocytic Ca^2+^, based on the lack of a vasodilatory response upon selective chemogenetic activation of astrocytes and on the preservation of arteriole dilatation upon sensory stimulation in IP_3_R2-KO mice (Nizar et al., 2013; Takata et al., 2013; Bonder and McCarthy, 2014). However, it should be noted that chemogenetic activation of non-native receptors expressed in astrocytes may not reproduce the spatial and temporal features of a physiological Ca^2+^ response. On the other hand, the dogmatic view of a complete Ca^2+^ signaling abolition in IP_3_R2-KO astrocytes has been recently cracked thanks to the use of genetically encoded calcium indicators (GECIs) of the GCaMP family, which allow to capture Ca^2+^ signals in the whole astrocyte territory (Shigetomi et al., 2013). Residual Ca^2+^ signals were indeed revealed through GECIs in astrocytes from IP_3_R2-KO mice at the level of thin processes (Srinivasan et al., 2015; Agarwal et al., 2017; Okubo et al., 2019) and in endfeet (Del Franco et al., 2021).

GECIs allowed to address also the other major criticism moved against a role for astrocyte Ca^2+^ in NVC, related to the timing of glial responses to neuronal activation, often reported delayed of seconds with respect to both neuronal activation and vasodilatation onset (Nizar et al., 2013; Bonder and McCarthy, 2014; Tran et al., 2018). While a pioneering study revealed a subset of fast responses to sensory stimulation also with the chemical indicator Oregon Green BAPTA-1 (Winship et al., 2007), later works supported this finding with more reliable, selective expression of cytosolic GCaMP in astrocytes. Ca^2+^ increases upon physiological neuronal stimulation were indeed observed in astrocytic processes before dilatation in the olfactory bulb (Otsu et al., 2015), in the retina (Biesecker et al., 2016) and in the SSCx (Lind et al., 2018). The membrane-bound probe Lck-GCaMP6f proved to be the more suitable to capture fast Ca^2+^ responses in astrocytic processes upon whisker stimulation in awake mice, also from IP_3_R2-KO animals (Stobart et al., 2018). In most cases, Ca^2+^ microdomains exhibiting fast onset were a subset of all astrocytic responses occurring upon neuronal activation. An important conclusion that we can derive from these and other experimental works [see, for example, (Institoris et al., 2022)] is that astrocytes display both fast and delayed responses during functional hyperemia, most likely playing different roles in initiating and maintaining vasodilatation. Many of the studies concluding that astrocyte response is too slow to be involved in vasodilatation onset present astrocyte response as an average time course of all Ca^2+^ signals recorded in processes or endfeet, thus diluting fast signals, whereas a more correct approach requires isolating the subset of fast responses to characterize their properties.

## Arterioles vs capillaries

An additional argument of dispute in the field arose in relation to the initiation site of vasodilatation, both in terms of cortical depth and vessel order. Since capillaries are not enwrapped in SMCs as arterioles, their active involvement in CBF has long been debated. Accordingly, the first studies investigated dilatation mostly at the level of penetrating arterioles, following them from the pial surface to the first cortical layers in both *ex vivo* and *in vivo* preparations. However, several reports demonstrate that the presence of contractile pericytes confers to capillaries the ability to regulate CBF in response to neuronal activity (Biesecker et al., 2016; Mishra et al., 2016; Khennouf et al., 2018). Most importantly, although vessels of all branching orders show dilatation in response to sensory stimulation, first order capillaries dilate before penetrating arterioles (Hall et al., 2014; Cai et al., 2018). Vasodilatation propagates then to upstream arterioles (and downstream into the capillary bed), most likely through retrograde electrical signaling in the endothelium. Indeed, since capillary endothelial cells express Kir channels, they can sense extracellular K^+^ released by astrocytes and neurons and transmit hyperpolarization along the vascular tree (Longden et al., 2017). The vasodilatation occurring in the capillary bed was estimated to produce 84% of the blood flow increase evoked by neuronal activity (Hall et al., 2014). Research studies investigating astrocyte role in the initiation of NVC should thus focus on the capillary level, and indeed recent reports show that astrocytes are involved in capillary- but not in arteriole-dependent control of CBF in the SSCx (Mishra et al., 2016) and in the retina (Biesecker et al., 2016). The former study nicely investigated the molecular pathways involved in NVC at the different vascular compartments. Vasodilatation evoked in capillaries was shown to depend on astrocyte Ca^2+^ signaling mediated by P2X1 purinergic receptors, followed by AA synthesis through phospholipase D and COX-1-dependent production of PGE2 ultimately acting on EP4 receptors (Figure 1B). The purinergic pathway mediating capillary vasodilatation was confirmed *in vivo* upon sensory stimulation (Mishra et al., 2016). The apparent conflict with respect to the previously described role of mGluRs in mediating astrocyte response to neuronal activity can be explained with developmental changes in receptor expression profiles in astrocytes (Sun et al., 2013). Conversely, the vasodilatory response evoked in arterioles was independent of these pathways and it relied instead on the activation of N-methyl-D-Aspartate receptors (NMDARs) and nitric oxide synthase (NOS), suggesting a prominent role of nitric oxide (NO) in the regulation of this vascular compartment. Of note, NMDAR-dependent NO synthesis has been reported not only in neurons but also in endothelial cells (Lu et al., 2019). While the involvement of NO in NVC has been well documented in different brain regions, it is commonly accepted that this molecule exerts in the cortex a modulatory and permissive rather than a direct role in NVC, by favoring vasodilatation through a reduction in the synthesis of the vasoconstrictor 20-HETE, while it directly evokes dilatation in the cerebellum (Attwell et al., 2010; Lourenço and Laranjinha, 2021).

## How to study astrocyte role in NVC

**FIGURE 2:**
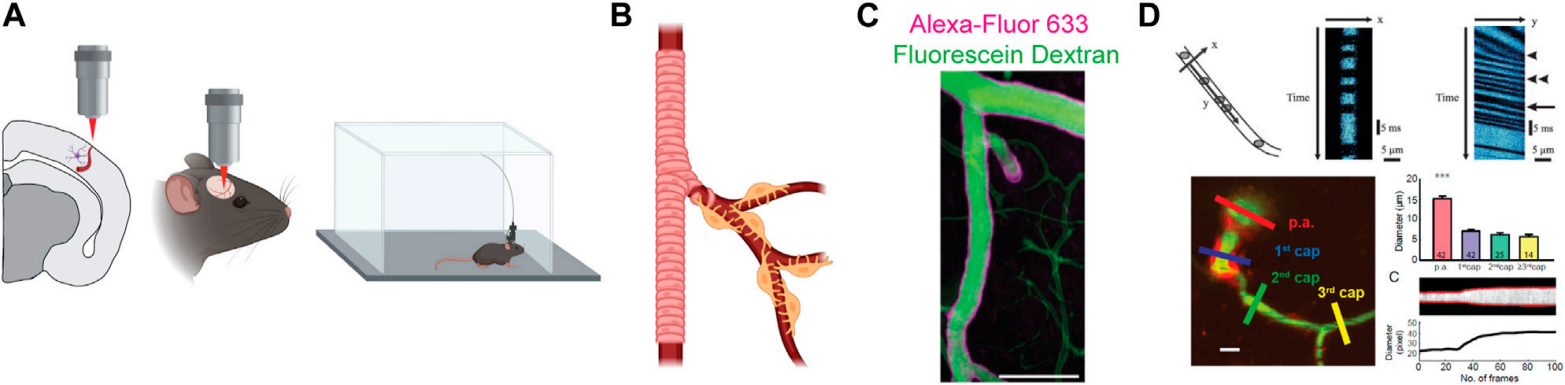
Methodological approaches to study NVC in mice. **(A)** Schematic view of different experimental approaches, from slice experiments to freely moving animals with miniscopes. **(B)** Distinction between arteriole, covered by smooth muscle cells, and capillary, covered instead by perycites. **(C)** Examples of two fluorescent dyes to label blood vessels. Alexa-Fluor 633 (magenta) selectively labels arterioles while fluorescein dextran (green) labels the lumen of both arterioles and capillaries. Image adapted from Shen et al., 2012. **(D)** Analytical approaches to measure NVC. Top, measurement of RBCs velocity. Adapted from Chaigneau et al. (2003). Bottom, measurement of vessel diameter. Adapted from Cai et al., 2018.

When facing the study of NVC, a fundamental aspect to be considered is the methodological approach (Figure 2). In this context, imaging techniques are an indispensable tool to properly analyze and detect regional changes in CBF. For the purpose of this review, we will focus on the applications related to two-photon laser scanning microscopy (2P-LSM), that allow us to follow both Ca^2+^ signaling in brain cells and vascular responses. A widely used approach couples the use of blood vessel labeling with GECIs to image neuron, astrocyte or mural cell activity. To quantify functional hyperemia, the most used parameter is the change in vessel diameter, usually extracted from imaging data after correction for movement artifacts in the xy plane. This measurement can also be applied to transmitted light images. Another possibility is to measure red blood cells (RBCs) velocity, which increases during functional hyperemia, an approach particularly useful for small capillaries in which assessing diameter changes is less reliable. Both approaches, for instance, have been used in (Tran et al., 2018).

Part of the controversies related to the vascular site of dilatation onset—arterioles vs capillaries—probably originate from a poor agreement on what to consider capillary or arteriole (Iadecola, 2017). Arterioles are wrapped by a continuous layer of SMCs while capillaries have sparse pericytes of different morphologies (Attwell et al., 2016; Hartmann et al., 2022). Researchers can thus take advantage of genetic mouse models with selective labeling of mural cells (Hill et al., 2015) to discriminate between arterioles and capillaries. An alternative strategy is the use of the artery-specific dye Alexa Fluor 633, that has been shown to selectively bind elastin fibers in arterial walls (Shen et al., 2012). A common strategy to label the lumen of cerebral vasculature involves tail-vein or retro-orbital injection of dextran-based dyes in blood plasma. In this case, the identification of vessel type is based on diameter criteria, with capillaries defined as vessels with an inner diameter of less than 10 µm (Attwell et al., 2016), or on the basis of the branching order, with the arteriole being the zero order and capillaries the successive orders (Zambach et al., 2021).

Functional hyperemia can be studied in both *in vivo* and *ex vivo* preparations. The first works on the role of astrocytes in NVC have been obtained mostly from brain slice preparations (Zonta et al., 2003a; Filosa et al., 2006; Gordon et al., 2008), which represent a widely used approach to study the molecular mechanisms of NVC due to the easy application of pharmacological compounds. However, the absence of a physiological synaptic activity and the artificial supply of energy substrates, i.e., oxygen and glucose, necessarily affect vasomotor activity (Gordon et al., 2011). Furthermore, the lack of a basal tone due to the absence of both intraluminal flow and intravascular pressure requires overcoming this limitation, through pretreatment of slices with constrictor agents (Filosa et al., 2006; Mishra et al., 2016) or cannulation of penetrating arterioles (Kim and Filosa, 2012). Nowadays, technical advancements allow to perform 2P-LSM experiments also *in vivo* preparations. Due to optical limitations and surgical complexity, these experiments have been classically performed in superficial cortical layers, although specific surgeries for hippocampal region or the use of GRIN lenses open new perspectives to study NVC in deeper brain regions (Gu et al., 2014; Chien et al., 2021). *In vivo* experiments led in some cases to different conclusions with respect to *ex vivo* studies. Since most studies are performed in anesthetized mice, an important confounding factor could be the use of anesthetics, which exert deleterious effects on both astrocyte Ca^2+^ activity (Thrane et al., 2012) and vascular reactivity (Tran and Gordon, 2015). For these reasons, raising the bar to an *in vivo* awake experimental approach avoids this possible source of controversies. NVC can be studied *in vivo* upon direct stimulation of brain activity by sensory stimulation (Institoris et al., 2022), or upon specific cell type stimulation. Over the past decades, optogenetics has been established as one of the most powerful ways to drive the activation of specific brain cells (Deisseroth, 2015). In the case of NVC research, caution is mandatory since a recent study proved that light *per se* induces vasodilatation in the neocortex and in the olfactory bulb by directly affecting Ca^2+^ activity in arteriolar SMCs (Rungta et al., 2017). Therefore, the use of optogenetics in this field requires proper control experiments to avoid artifacts. Another way to tackle the specific contribution of different cell types is to employ genetic tools to modulate specific intracellular pathways. For astrocytes, one of the most used approach is the chemogenetic activation *via* designer receptors exclusively activated by designer drugs (DREADDs), which increase intracellular Ca^2+^ specifically in astrocytes by using an exogenous compound, typically clozapine N-oxide (CNO) (Adamsky et al., 2018). Conversely, other tools were recently developed to attenuate astrocyte Ca^2+^ activity, such as CalEx and iβark, which hamper Ca^2+^ signals in astrocytes respectively by extruding Ca^2+^ or by attenuating G_q_ GPCR signals (Yu et al., 2018; Nagai et al., 2021). In order to apply these tools in NVC research, control experiments with appropriate GECIs are needed to verify their efficacy in silencing localized Ca^2+^ activity in astrocyte processes and endfeet.

Finally, technological advancements allow the study of brain dynamics in freely moving animals through the use of miniaturized microscopes, thus avoiding the need for the head restrained configuration required for awake 2P-LSM experiments. Recently, a first application of this approach has been shown in the context of cortical injury (Lin et al., 2022). Studies in this direction will probably shed new light on NVC mechanisms in different brain regions coupled with different behavioral contexts and possibly also in pathological conditions.

## NVC and pathology

### Ischemic stroke

Ischemic stroke occurs when a blood vessel is blocked by a clot or embolus. Poststroke patients show decreased NVC that lasts up to a decade after the initial infarct (Krainik et al., 2005; Lin et al., 2011) and different experimental evidences indicate dysregulation of vascular control (Figure 1C). *Ex vivo* models of cerebral ischemia based on the application of hypoxic conditions revealed rapid depolarization of neuronal and glial cells resulting from changes in the extracellular ion concentration and resembling spreading depression (Müller and Somjen, 2000). In the ischemic core, acute ATP depletion indeed impairs the activity of Na^+^/K^+^-ATPase and opens ATP-sensitive potassium channels, resulting in substantial [K^+^]_o_ increase (Dreier and Reiffurth, 2015; EbrahimAmini et al., 2022). While a moderate elevation of extracellular K^+^ is a powerful vasodilatory signal due to its hyperpolarizing effect, [K^+^]_o_ exceeding 20 mM can result in a diffuse constriction of local vasculature by direct depolarization of mural cells (Filosa et al., 2006).

Beside [K^+^]_o_, other vasoactive players can influence the vascular response after stroke. A recent work employed transient middle cerebral artery occlusion (MCAO) as a stroke model to investigate the capillary hyperemic response in brain regions outside the ischemic core. The results reveal an impairment in NVC in the peri-infarct cortex 1 day after MCAO, which could be reverted by inhibiting the synthesis of the vasoconstrictor 20-HETE. Consistently, cortical 20-HETE levels were increased after MCAO, in agreement with observations from stroke patients (Li Z. et al., 2021). The dysregulation of 20-HETE levels most likely reflects a decrease in NO production, which affects the balance of the vasoactive compounds derived from AA by reducing the established inhibitory action of NO on 20-HETE synthesis.

### Alzheimer’s disease

Alzheimer’s disease (AD) is the most common neurodegenerative disorder worldwide, characterized by progressive cognitive impairment and memory loss. Common hallmarks are extracellular amyloid-β (Aβ) oligomers and plaques, and intraneuronal accumulation of neurofibrillary tangles.

Neurovascular dysfunction is an early event in the pathogenesis of AD, involved in a positive feedback loop described by the two-hit vascular hypothesis, in which vascular abnormalities favor AD pathogenesis and Aβ burden contributes to worsen CBF function and regulation (Zlokovic, 2011; Zhu et al., 2022). Along with neurons, the other cells composing the neurovascular unit can degenerate or alter their signaling pathways in AD (Figure 1D). Consistent with the central role of capillaries in NVC, pericyte loss in AD mouse models contributes to NVC impairment, besides inducing BBB breakdown (Sagare et al., 2013; Kisler et al., 2017). In addition, accumulation of Aβ in the proximity of pericytes induces reactive oxygen species (ROS) production, which evokes the release of the strong vasoconstrictor endothelin-1 (ET-1) (Nortley et al., 2019).

The accumulation of Aβ oligomers around cerebral vasculature has been known since the 70s (Mandybur, 1975). Recent electron microscopy studies revealed that vascular Aβ develops in ring-like structures around vessels inducing a physical displacement of astrocyte endfeet, thereby reducing the ability of astrocytes to regulate vascular tone. In these conditions, also mural cell response to vasoactive compounds is dampened, presumably due to the stiffness of Aβ rings (Kimbrough et al., 2015). AD development is also associated with astrocyte reactivity, resulting in transcriptional and morphological changes in astrocytes (McConnell et al., 2019). It is worth mentioning that transcriptome analysis in astrocytes from AD patients revealed expression changes in 32 genes associated with Ca^2+^ signaling (Simpson et al., 2011). Consistently, early astrocyte calcium dysfunction is found in different AD mouse models, with both hyperactivity and hyporesponsiveness reported (Kuchibhotla et al., 2009; Lines et al., 2022; Åbjørsbråten et al., 2022; Lia et al., 2023). Along this line, an attenuated astrocyte endfeet response to neuronal stimulation has been recently suggested to contribute to NVC impairment in APP mice, in which reactive oxygen species (ROS) were also involved (Li L. et al., 2021).

Another crucial NVC pathway affected in AD models relates to the role of K^+^ channels in initiating and propagating the hyperpolarization that drives vasodilatation. Recent studies on different AD mouse models report a reduction in the activity of endothelial Kir 2.1 channels, resulting in altered vascular responses to extracellular K^+^ (Hakim and Behringer, 2020; Li L. et al., 2021; Mughal et al., 2021). In the context of AD, the reduction in NVC has been linked also to a deficiency in tissue plasminogen activator (tPA), a serine protease which is physiologically implicated in the release of NO upon NMDAR activation. The interaction of tPA with NMDARs is indeed necessary for the increase of NOS activity induced by NMDAR activation (Anfray et al., 2020), and tPA-KO mice present a reduced CBF upon whisker stimulation (Park et al., 2008). Interestingly, tPA activity is reduced in both AD human brain samples (Angelucci et al., 2022) and in a mouse model of AD, where an increased tPA inhibition has been associated with the attenuation of NVC following whisker stimulation (Park et al., 2020), consistently with an impairment of NO pathway. Lower expression of NOS was also found in the hippocampus of patients affected by AD, while no differences were reported in the cerebellum, in line with the fact that this region is affected at later stages of the disease (Liu et al., 2014; DeTure and Dickson, 2019).

## Conclusion

At the end of this journey across 20 years of NVC history and research, we believe that the controversies raised in this field stimulated a positive scientific debate that ultimately refined the comprehension of the underlying mechanisms. Science improves our knowledge as long as we are both open to doubts and rigorous in performing experiments dealing with them, underlining the importance of choosing the appropriate tools to study NVC.

While this review focuses on the role of astrocytes in NVC, it is not meant to claim that these cells are the exclusive players involved. Similarly, the main pathways presented here do not exclude parallel mechanisms, which can have a modulatory or a primary role in different brain regions or in specific tissue conditions, with the role of NO derived from neurons or endothelial cells being an example of this concept. It is highly conceivable that the brain has evolved a redundancy of mechanisms to secure NVC through the involvement of different cell types and different molecular pathways. The ongoing research is expected to further deepen our understanding of these mechanisms, and hopefully to provide new strategies to face neurovascular impairment in different brain diseases.
